# To What Affections of the Lungs Does Bronchitis Give Origin?

**Published:** 1860-12

**Authors:** 


To What Affections of the Lungs does Bronchitis Give Origin?—An Essay, by Daniel D. Slade, M.D., of Boston, for which a Prize of $100, was awarded by a Committee of the Massachusetts Medical Society.

This is a very interesting Essay of 75 pages, printed on good paper, and in good legible type. It presents a fair summary of what is known concerning the subject on which it treats; but claims no novel discoveries or important additional facts. We quote the closing pages of the Essay, which will give our readers an idea of the topics discussed, and the conclusions drawn by the author:

"In the preceding pages, we have endeavored to point out the immediate and remote effects of bronchitis, as shown more particularly by the *pathological* states of the lung. We first directed attention to the effect of inflammation upon the lining membrane of the bronchi, and their secretions, and the consequent effects upon the auscultatory phenomena of the chest. We then spoke of death from apnœa, the result of sudden and abundant effusion of the inflammatory secretion, or of the plugging up of one or more of the principal bronchi. We proved that all the phenomena exhibited by the physical signs of bronchitis were in perfect accordance with the anatomical appearances, which we described.

"Next, we considered the direct effects of the obstruction of the bronchi upon the adjacent pulmonary tissue, leading to that peculiar condition, collapse of the lung, a lesion which has but lately been properly understood, having been heretofore considered and described as a form of pneumonia. The history of this affection we discussed at some length—commenting upon the light which a knowledge of it had thrown upon the pathological condition of the lung, particularly in childhood.

"We next considered the causes of this lesion—and whether obstruction of the bronchi, without some deficiency in the respiratory power, was sufficient to bring it about. We gave the views of several observers on this point, and the results of experiments on animals—and having discussed the relative effects of inspiration and expiration, in their power to get rid of bronchial obstructions, as well as the mechanical condition, conducing to the production of collapse, to be found in the air-tubes themselves, we arrived at the following conclusions:

"That the production of collapse of the lung is due—first, to the existence of mucus in the bronchi, which is the more liable to produce collapse in proportion as it is tenacious. Second, to weakness or inefficiency of the inspiratory power, however it may be caused. Third, to inability to cough or to expectorate, and thus remove the obstructing mucus.

"Bronchitic collapse of the lung occurring under two different forms, we gave the anatomical appearances which they present, observing also, that the disease offered the same characteristics in the adult as in the child.

"The question of bronchial abscess next occupied us. We considered its pathology and relation to bronchitic collapse and, the views of several observers on this point.

"The diagnostic symptoms of collapse having been given, we considered, whether this condition being once established, the lung could be restored to its normal condition—a consideration which led to the question of the function belonging to the muscular fibres of the air-tubes.

"Does bronchitis give rise to true pneumonia, lobular as well as lobar? We presented the views of several authors on this point—as also the anatomical appearances of partial (lobular) pneumonia.

"We next passed on to the secondary and more permanent lesions of the lung—the result, for the most part, of bronchitic collapse. We said that this pulmonary lesion led to atrophy of the lung—which we fully considered, giving the observations of Dr. Gairdner, and others, on this point, and on the pulmonary concretions which are not unfrequently found in the midst of atrophied lung.

"Next in order, as secondary effects of bronchitis, we discussed the important subject of vesicular emphysema. Describing the nature of this lesion, we considered at some length the conflicting theories which have been offered to account for its development. We endeavored to show that the theory of Laennec and others, which would ascribe the origin of emphysema to forced *expiration*, could not be supported, reasoning on the mechanical incapability of the act, but that the experiment of M. Groux, upon himself, would seem to decide otherwise; so that we are forced to admit that vesicular emphysema *may* be produced by the expiratory act.

"The *inspiratory* theory of Dr. Williams and others, we attempted to prove, approached the truth, but did not cover the entire ground. Reasoning from the fact that the inspiratory power of the chest is exactly limited by its capacity, it is obvious that inspiratory force can no more distend the air-cells so as to produce emphysema, than it can do so in perfect health. Another condition is therefore necessary to the perfection of the theory, and this is to be found in partially diminished bulk, or, in other words, pulmonary collapse or permanent atrophy of some portion of the lung.

"These observations are based not only upon what the anatomical appearances teach, but upon the peculiarities which are presented by the 'emphysematous chest,' and the relation which it bears to pulmonary emphysema.

"Certain pathological alterations of the bronchi, the contraction, obliteration, as well as the dilatation of the vessels, as secondary results of bronchitis, were next attended to. The forms, causes and changes in the lung due to these conditions, were spoken of.

"The nature of asthma, and its relation to bronchitis, were discussed. Having again spoken of the de-obstruent action of the air-tubes, we said that attacks of asthma, especially that which is termed 'Humoral,' were undoubtedly owing in many cases to a spasmodic derangement of the muscular fibres of the bronchi, whereby a great accumulation of mucus took place. Connecting these two phenomena, it is obvious that if this removal of the mucus depends in a healthy condition upon the regular action of these fibres, its accumulation must accompany any derangement of that action.

"In our remarks upon the relation of bronchitis to phthisis, we said that observation and experience have now conclusively shown that this affection is to be considered as a cause of phthisis *only when a predisposition exists;* that there was in reality very little or no foundation for the widely-spread opinion that bronchitis is a frequent and direct cause of phthisis.

"We closed with some general observations upon the etiology and prophylaxis of the disease."

